# Novel ω-conotoxins from *C. catus* reverse signs of mouse inflammatory pain after systemic administration

**DOI:** 10.1186/1744-8069-9-51

**Published:** 2013-10-20

**Authors:** Mahsa Sadeghi, Swetha S Murali, Richard J Lewis, Paul F Alewood, Sarasa Mohammadi, MacDonald J Christie

**Affiliations:** 1 Discipline of Pharmacology, University of Sydney, Sydney, NSW 2006, Australia; 2 Institute for Molecular Bioscience, University of Queensland, St Lucia, QLD 4072, Australia

**Keywords:** Calcium channel, Conotoxin, Inflammatory pain

## Abstract

**Background:**

Antagonists of N-type voltage-gated calcium channels (VGCC), Ca_v_2.2, can manage severe chronic pain with intrathecal use and may be effective systemically. A series of novel ω-conotoxins that selectively inhibit N-type VGCCs was isolated from *Conus catus.* In the present study, the potency and reversibility of ω-conotoxins CVID, CVIE and CVIF to inhibit N-type calcium currents were investigated in mouse isolated dorsal root ganglion (DRG) neurons. The systemic potency of each ω-conotoxin to reverse signs of mouse chronic inflammatory pain was also compared.

**Results:**

In DRG neurons, the rank order of potency to inhibit N-type calcium currents was CVIE > CVIF > CVID. After subcutaneous administration, CVID and CVIE, but not CVIF, partially reversed impaired weight bearing in mice injected with Freund’s complete adjuvant (CFA) three days prior to testing. No side-effects associated with systemic administration of ω-conotoxins were observed.

**Conclusions:**

The present study indicates a potential for CVID and CVIE to be developed as systemically active analgesics with no accompanying neurological side-effects.

## Introduction

Antagonists of the N-type voltage-gated calcium channel (VGCC), Ca_v_2.2, are potential therapeutics for the treatment of pain. ω-Conotoxins are highly selective N-type VGCC inhibitors isolated from the venom of cone snails (genus *Conus*) [1]. After intrathecal administration, they act to prevent conduction of nociceptive signals from the peripheral to the central nervous system by inhibiting neurotransmitter release from primary afferent nerve terminals in the dorsal horn of the spinal cord. The first FDA approved ω-conotoxin MVIIA (SNX-111, ziconotide or Prialt) is administered intrathecally for management of severe and chronic pain in patients who are intolerant or refractory to the other treatments [2]. However, the therapeutic index of intrathecally administered Prialt is small, producing severe side effects in many patients at therapeutic doses [3,4] and in animal models [5-7].

ω-Conotoxins are administered intrathecally to selectively target Ca_v_2.2 channels in the spinal cord. ω-Conotoxin MVIIA is unsuitable for parenteral administration because it produces serious side effects including cardiovascular and autonomic dysfunction [6,8,9]. On the other hand, the invasive surgery, high cost of implantation of pump, and the risk of infection limit spinal administration [8,10]. More recent studies have suggested that other ω-conotoxins, such as CVID, can produce some pain relief following parenteral administration with a wider therapeutic window than MVIIA [8]. Intravenous injection of ω-conotoxin CVID produced dose-dependent antihyperalgesic effects without serious cardiovascular side effects [8]. The mechanism responsible for the superior therapeutic index of CVID versus GVIA and MVIIA is not yet known although it could be due to its higher selectivity for N-type VGCCs over the P/Q-type (Ca_v_2.1) [5,6,11,12]. However, other biophysical mechanisms of interaction of CVID with Ca_v_2.2 [12], different biodistribution or metabolism may also be responsible.

We recently identified a novel series of ω-conotoxins from *Conus catus*[12]. Importantly, CVIE and CVIF potently and reversibly inhibited Ca_v_2.2 channel currents in a voltage-dependent manner and reversibly inhibited excitatory monosynaptic transmission in the dorsal horn of the spinal cord. In contrast, CVID, MVIIA and GVIA produced irreversible inhibition [13,14]. CVIE and CVIF produced similar but reversible inhibition of mechanical allodynia in a nerve-injured rat model after intrathecal administration [12]. The combination of high potency, selectivity and reversibility of Ca_v_2.2 block might contribute to improved efficacy or a wider therapeutic index of ω-conotoxins after intrathecal and potentially parenteral administration.

In the present study, we investigated the potency and reversibility of CVIE and CVIF in isolated sensory neurons dissociated from mouse dorsal root ganglion neurons and determined if systemic administration of these ω-conotoxins produced greater inhibition of inflammatory pain-related behaviours when compared to CVID in mice.

## Results

### Inhibition of whole-cell I_Ca_ by ω-conotoxins in isolated DRG neurons

**Figure 1:**
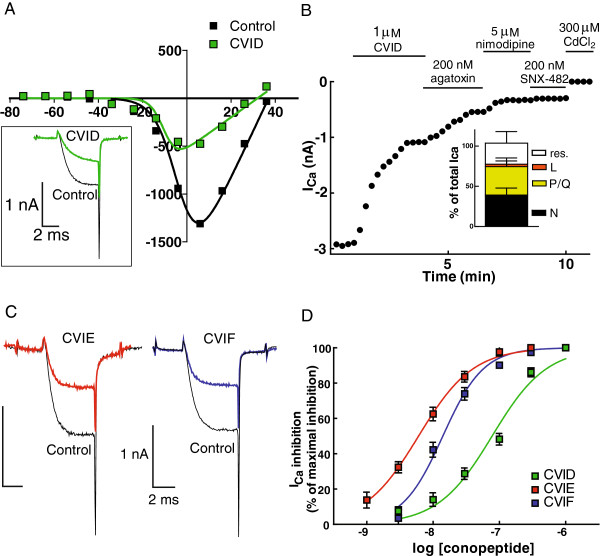
**Inhibition of VGCCs in DRG neurons by ω-conotoxins. A**. Current–voltage (I-V) curve showing the block of high-voltage activated I_Ca_ by 300 nM CVID. *Inset,* representative recording of I_Ca_, with the current trace following block by CVID shown in green. **B**. Time-plot of inhibition of HVA I_Ca_ by specific blockers for different calcium channel subtypes, i.e. CVID for N-, ω-agatoxin IVA for P/Q-, nimodipine for L-, SNX-482 and CdCl_2_ for I_Ca_ resistant (res.) to all other blockers . *Inset,* proportions of the different subtypes shown as percentages present in mouse DRG neurons. **C**. Representative recordings of I_Ca_ showing block by ω-conotoxins CVIE and CVIF. **D**. Concentration-response curves for each ω-conotoxin.

Robust inhibition of total I_Ca_ was seen with CVID (Figure 1A). As shown in Figure 1B, CVID inhibited about 40% of the total I_Ca_, ω-agatoxin IVA, approximately 30%, nimodipine 5%, and the residual current was blocked by SNX-482 and CdCl_2_. As previously reported in mouse trigeminal ganglion neurons [15] using other N-type VGCC antagonists, N-type current inhibited by each ω-conotoxin contributed to approximately 50% of the total I_Ca_ (Figure 1C). As shown in Figure 1B, Agatoxin IVA inhibited approximately 30% of the total I_Ca_ representing the contribution of P/Q-type channels (Ca_v_2.1), nimodipine inhibited 4% representing the L-type, the blocker of R-type channels (Ca_v_2.3), SNX-482, was without effect and residual I_Ca_ was blocked by CdCl_2_ (50 μM). The nature of the residual Ca current is unknown but could represent R-type channel variants known to be insensitive to SNX-482 [16], as well as other Ca_v_ currents.

When the concentration-response curves were plotted (Figure 1D), CVIE was found to be the most potent at I_Ca_ inhibition, more than ten-fold compared to CVID (P < 0.001 using two-way ANOVA with Bonferroni’s post-test), which was the least potent. Inhibition parameters are shown in Table 1.

**Table 1:** **Inhibition parameters of the three ω-conotoxins on I**_**Ca **_**recorded from isolated DRG neurons**

**Peptide**	**CVID**	**CVIE**	**CVIF**
Maximal Inhibition of I_Ca_ (% of total)	52 ± 2	45 ± 4	47 ± 6
Log [IC_50_]	-7.12 ± 0.04	-8.22 ± 0.03	-7.85 ± 0.03
Recovery from block (% of inhibition, 10 min)	-1 ± 5	0 ± 3	24 ± 10
Peak inhibition vs. recovery at 10 min (*t*-test)	P = 0.3	P = 0.2	P < 0.05

No significant difference was observed in the maximal inhibition by any of the peptides (P = 0.52 with one-way ANOVA). Differences between the concentration-responses of the three peptides were tested using two-way ANOVA (P < 0.0001) and followed up with Bonferroni’s post-test. The IC_50_ of CVID was significantly less than that of CVIE and CVIF (P < 0.001 for both comparisons), but no difference was found between CVIE and CVIF (P > 0.05). Hill slopes of all three concentration-response relationships were not significantly different from 1. Recovery from block was tested using paired *t*-test, and significant recovery was observed only from inhibition by CVIF (P < 0.05).

**Figure 2:**
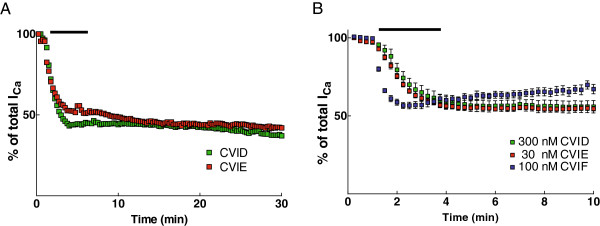
**Reversibility of inhibition of VGCCs by ω-conotoxins. A**. Representative time plots from two neurons showing block of I_Ca_ (normalised to maximum I_Ca_) by CVID and CVIE, followed by washout. Note that no recovery was observed up to 30 minutes of washout of the peptides. **B**. Summary timeplots showing recovery from I_Ca_ block following washout of the different conopeptides at 10 minutes. Partial recovery was seen following washout of CVIF and no recovery was observed after application of CVID and CVIE.

We then examined the reversibility of I_Ca_ inhibition by the different conotoxins. As shown in Figure 2A, no recovery was seen from CVID and CVIE block even after 30 min of recording. Figure 2B shows the summary of recovery from the different conotoxins over 10 min. CVID and CVIE were completely irreversible and CVIF was partially reversible with recovery of up to 24% (see Table 1).

### Development of hyper-responsiveness: incapacitance versus automated von Frey

**Figure 3:**
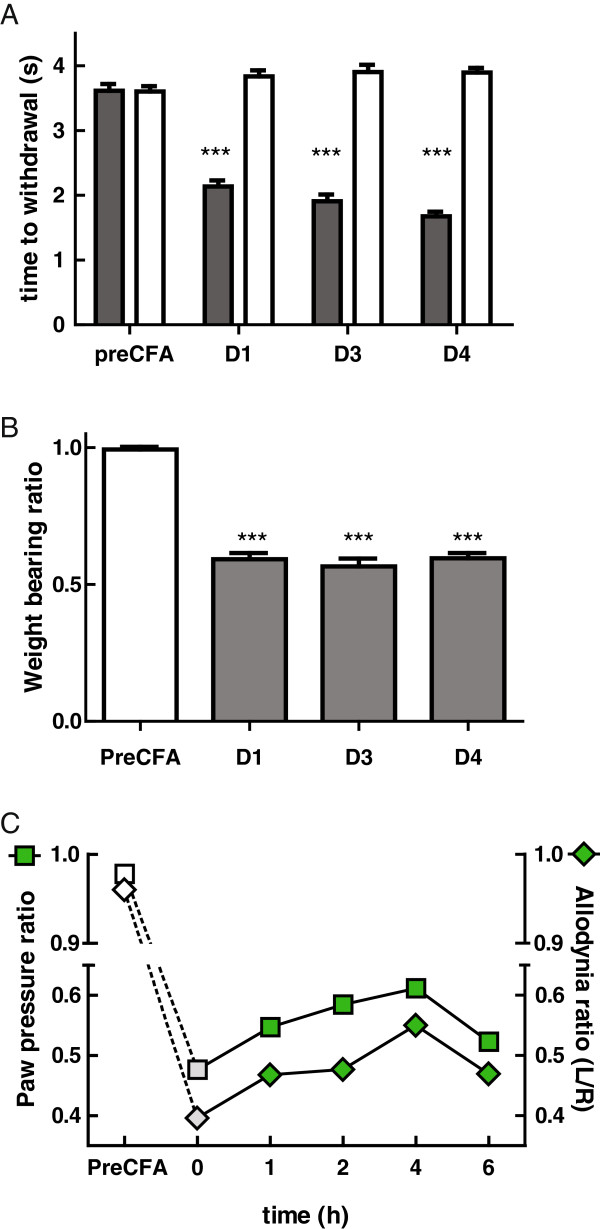
**CFA-induced mechanical thresholds using von Frey filaments and incapacitance. A**. Time to withdrawal measured using automated von Frey testing showed that both paws showed equal latency before CFA injection. Following injection, the time to withdrawal was significantly reduced in the injected paw compared to the contralateral paw, and the effect remained up to four days following CFA injection. **B**. Incapacitance testing was used to measure the ratio of weight-bearing between the two paws. This ratio went from 1 before CFA injection, indicating equal weight-bearing, to close to 0.5 following injection, indicating preference of the contralateral paw. This effect was measurable up to four days following CFA injection. **C**. Comparison of automated von Frey and incapacitance testing showed similar development of allodynia, which could be partially reversed by administration of CVID. No differences were seen between the two methods, therefore incapacitance testing was used in subsequent experiments in the interest of time.

As shown in Figure 3, automated von Frey (Figure 3A) and incapacitance (Figure 3B) testing revealed hyper-responsiveness of the inflamed hindpaw for at least 4 days following injection of CFA. In order to determine if incapacitance and automated von Frey testing produced similar outcomes, responses to von Frey filaments were correlated in groups of mice injected with CFA (n = 14) or saline (n = 14), 4 days After injection. Responses were highly correlated (R^2^ = 0.94, P < 0.001). In a pilot study of 4 animals, 2 mg/kg CVID was injected 3 days after induction of inflammation and hyper-responsiveness was assessed using both measures. Since both measures appeared to be sensitive to subcutaneous injection of CVID (Figure 3C), we chose the simpler incapacitance measure for the remainder of the study.

### Time- and dose response relationships for ω-conotoxins in vivo

#### CVID

**Figure 4:**
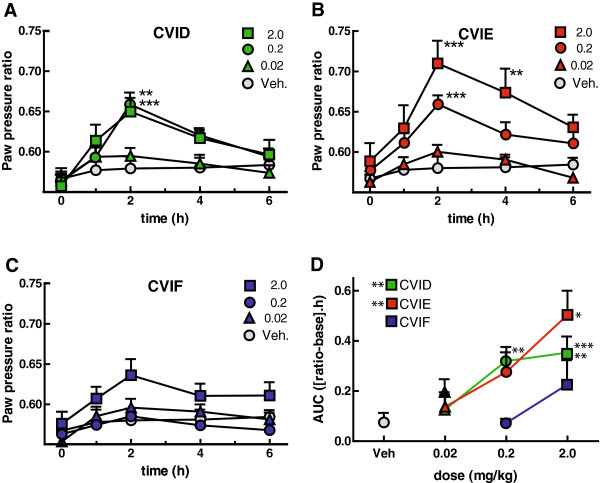
**Time-course and dose-dependence of reversal of allodynia by systemic ω-conotoxins. A-C**. Systemic ω-conotoxins partially reverse CFA-induced incapacitance. Effects of CVID, CVIE and CVIF respectively at doses of 0.02, 0.2 and 2 mg/kg on allodynia following CFA injection. **D**. Area under the curve measurements corrected for baseline with vehicle show that CVID and CVIE were the most effective at reversing mechanical alldoynia, with CVID reaching a ceiling effect at 0.2 mg/kg which was not seen with CVIE.

The time-response curves for each dose of CVID and vehicle are shown in Figure 4A. CVID produced reversal of incapacitance, with significant reversal at the peak time of action (2 h) for both 0.2 and 2.0 mg/kg doses. The response to CVID appeared to reach a ceiling at 0.2 mg/kg. Overall significance of AUC data was also achieved for 0.2 and 2.0 mg/kg (Figure 4D).

#### CVIE

The time-response curves for each dose of CVIE and vehicle are shown in Figure 4B. CVIE produced significant reversal of incapacitance at the points indicated. For CVIE, 2.0 mg/kg was clearly the most effective dose. It should be noted that unlike CVID, CVIE did not appear to reach a ceiling at the highest dose tested. Overall significance was achieved for AUC data at 2.0 mg/kg. Although CVIE appeared to produce greater maximal reversal of incapacitance than CVID, two factor ANOVA of AUC for CVID and CVIE dose–response data did not reveal any significant differences.

#### CVIF

The time-response curves for each dose of CVIF and vehicle are shown in Figure 4C. Although there was a trend for reversal of incapacitance at the highest dose, CVIF did not produce significant reversal of incapacitance.

#### Dose–response relationships

Figure 4D shows the AUC calculated for each animal with the corresponding pre-injection baseline subtracted. One-way ANOVA was used to test the difference between the dose and vehicle for each dose of each drug, and the significance is indicated in Figure 4D. To test whether there was a significant potency difference among peptides, a two-way ANOVA with all possible post-hoc contrasts using Bonferroni correction (excluding vehicle). CVIE was significantly more potent than CVIF at 2.0 mg/kg (P < 0.01) but not CVID at any dose.

#### Side effects

None of the animals exposed systemically to ω-conotoxins displayed any of the typical side effects usually observed after intrathecal administration [5-7]. No other overt signs were noted in animals injected with ω-conotoxins. Three vehicle injected animals displayed tail shaking and were not included in the analysis because their incapacitance scores were also unreliable. To determine if systemic ω-conotoxins produce any effects on motor coordination or performance, maximal doses (2 mg/kg) of each conotoxin on rotarod performance and grip strength was tested at the time of maximal effect (2 h) in a separate cohort of mice. As shown in Table 2, none of the conotoxins produced a significant effect on motor coordination or grip strength.

**Table 2:** Effects of the three ω-conotoxins on motor coordination tested on an accelerating rotarod and grip strength test using a grip strength meter

**Treatment/test**	**Rotarod performance**		**Grip strength**	
	**Time to fall (s)**	**End rate (rpm)**	**Peak tension (g)**	**Tension/weight**
Vehicle (n = 9)	39 ± 4.2	18 ± 1.4	130 ± 5.5	5.8 ± 0.3
CVID (n = 7)	40 ± 4.1	18 ± 1.4	121 ± 5.7	5.7 ± 0.3
CVIE (n = 7)	36 ± 4.1	17 ± 1.4	121 ± 6.9	5.5 ± 0.3
CVIF (n = 7)	36 ± 3.0	17 ± 1.0	121 ± 7.7	5.4 ± 0.4

All ω-conotoxins were tested in naïve mice at the maximal analgesic dose (2 mg/kg, s.c.) at the time of maximal analgesic effect (2 h). None of the ω-conotoxins produced any significant effect on ability of mice to perform on the rotarod or maintain grip strength (each parameter tested by one-factor ANOVA; all P > 0.6).

## Discussion

ω-Conotoxins selective for N-type calcium channel are an established class of therapeutics for the treatment of chronic pain and there is evidence that they have some efficacy with systemic administration. In this study we characterized the effects of two novel ω-conotoxins, CVIE and CVIF. All conotoxins selectively inhibited N-type calcium channels in mouse DRG neurons, consistent with a previous report in rat [12]. As expected, the maximal inhibition by these conotoxins was very similar to that of CVID, which also has high selectivity for N-type calcium channels [17]. The contribution of the other types of calcium channels was also determined using specific blockers for P/Q-type (ω-agatoxin IVA) and L-type (nimodipine), as well as CdCl_2_ to block the residual resistant current. The lack of effect of the R-type inhibitor, SNX-482, suggests that the Cd^2+^-sensitive residual current may be mediated, in part, by a variant of Ca_v_2.3 that is insensitive to this peptide [16]. Other channels may underlie this resistant current because a residual current is also present in Ca_v_2.3 knock-out mice [18]. These results confirm that N-type calcium channels contribute to approximately 50% of the total I_Ca_. We also found that CVIE was the most potent of the conotoxins examined, with a more than ten-fold greater IC_50_ than that of CVID, which was the least potent.

A potential determinant of the therapeutic window of ω-conotoxins is their reversibility because a high side-effect profile is seen with the irreversible ω-conotoxins, including MVIIA and CVID [19]. Therefore, we were interested in the reversibility of these ω-conotoxins *in vitro*. At -80 mV, CVID and CVIE were completely irreversible and CVIF was partially reversible, consistent with previous reports [12]. We did not observe any side effects following administration of all three peptides, indicating a potential for these conotoxins to be administered systemically. This also indicated that in our study, irreversibility *in vitro* did not produce behavioural side-effects *in vivo* following peripheral administration. The absence of side-effects in this study is also consistent with the report from Kolosov *et al.*[8] who reported much lower side-effect scores for intravenously administered CVID compared to MVIIA in rat. None of the ω-conotoxins produced obvious side effects or significant effects on motor coordination and grip strength but it will be important in future studies to determine the relative ability of CVID and CVIE to produce hypotension as this is a potential dose-limiting side effect in humans. The lack of neuronal side-effects following systemic administration of ω-conotoxins could be due to a differential expression of auxiliary subunits associated with the N-type calcium channel in sensory and autonomic neurons. It has been shown previously that recovery from CVIE and CVIF block is much greater in the presence of β2a subunits compared to β3 subunits [12]. Additionally, the presence of α2δ subunits significantly reduces the affinity of CVID for N-type calcium channels, while decreasing recovery from CVID block [20]. Bioavailability and distribution may also play a role, however, less is known about those factors at present.

When we compared the *in vivo* and *in vitro* concentration-responses of these peptides, our findings were quite unexpected. CVID and CVIE were most effective at reversal of pain, with the former reaching a ceiling effect. CVIE appeared to produce greater reversal of incapacitance at the highest dose but this was not significantly greater than CVID. This indicates that *in vivo*, CVID is more potent than CVIE, contrary to the results *in vitro*. It is possible that reversibility of the peptides would increase potency *in vivo.* However, we found no significant differences in reversibility between CVID and CVIE, suggesting that this explanation would only be plausible if reversibility differed greatly in peripheral target tissues other than DRG neurons. Distribution and bioavailability could be significant in determining the actions of CVID and CVIE *in vivo*, which is beyond the scope of the present study.

## Conclusions

The major finding of this study was that systemically administered ω-conotoxins CVID and CVIE reverse signs of inflammatory pain. Importantly, this was not accompanied by side-effects such as motor deficits or sedation. The *in vitro* potency and reversibility of all ω-conotoxins examined were not predictive of efficacy or presence of side-effects *in vivo*. This underscores the need for future studies to investigate factors such as bioavailability, distribution and tissue-specific effects of these peptides.

## Methods

Male C57 Bl6 mice (20–25 g, n = 177 for *in vivo* experiments, and 6–10 week-old, n = 20 for *in vitro* DRG neuron recordings) were used in this study. Animals were housed in groups of four to six with environmental enrichment on a 12 h/12 h light–dark cycle at 22 ± 2°C, with *ad libitum* access to food and water. All experiments were conducted according to protocols approved by the Animals Ethics Committee of the University of Sydney, Sydney, NSW, Australia which complies with the National Health and Medical Research Council 'Australian code of practice for the care and use of animals for scientific purposes’.

### Isolated DRG neuron preparation

DRG neurons were isolated from adult mice (6–10 weeks) as previously described [21]. Mice were anaesthetized with isofluorane (4% in air) and decapitated. Dorsal root ganglia (spinal levels L3 – L5) were removed and placed in ice-cold Hepes-buffered saline (HBS) containing (mm): NaCl, 154; KCl, 2.5; CaCl_2_, 2.5; MgCl_2_, 1.5; Hepes, 10; glucose, 10; pH 7.4 (NaOH), 330 ± 5 mosmol l^-1^. Ganglia were cut up with iridectomy scissors and incubated at 37°C for 15 min in oxygenated HBS containing 3 mg ml^-1^ collagenase and for 25 min in oxygenated HBS containing 1 mg ml^-1^ papain. The digestion was terminated with addition of HBS containing 1 mg ml^-1^ bovine serum albumin and 1 mg ml^-1^ trypsin inhibitor. Ganglia were washed free of enzyme and enzyme inhibitors with room-temperature HBS. Cells were dispersed by gentle trituration through decreasing bore, silanized Pasteur pipettes with fire-polished tips. The cells were plated onto plastic culture dishes and kept at room temperature in HBS. Cells remained viable for up to 10 h after dissociation.

### Electrophysiological recording from DRG neurons

Ionic currents from mouse DRG neurons were recorded in the whole-cell configuration of the patch-clamp method at room temperature (22 – 24°C) as previously described [21]. Dishes were continually superfused with HBS. For isolating I_Ca_, the extracellular solution contained (mM): 140 tetraethylammonium chloride, 2.5 CsCl, 2.5 CaCl_2_, 10 Hepes, 1 MgCl_2_, 10 glucose; pH 7.2 (with CsOH), 330 ± 5 mosmol l^-1^. The intracellular pipette solution contained (mM): 120 CsCl, 10 Hepes, 10 EGTA, 2 CaCl_2_, 5 MgATP, 0.2 Na_2_GTP, 5 NaCl; pH 7.3 (CsOH), 285 ± 5 mosmol L^-1^. Recordings were made using an EPC-9 patch-clamp amplifier and corresponding PULSE software from HEKA Electronik (Lambrecht/Pfalz, Germany). Currents were sampled at 20 – 50 kHz and recorded on hard disk for later analysis. Patch pipettes were pulled from borosilicate glass (AM Systems, Everett, WA, USA). The pipette input resistance ranged between 1.5 and 2.5 MΩ. The capacitance of individual cells was estimated by PULSE software by fitting an exponential to current responses to small rectangular voltage pulses and ranged between 10 and 50 pF. Series resistance was between 3 and 10 MΩ. Series resistance compensation of between 70% and 80% was used in all experiments. Capacitative transients were compensated automatically using a built-in procedure of the HEKA amplifier. Leak current was subtracted online using a *P*/8 protocol. Liquid junction potential of 4 mV was not corrected for.

Peak high voltage-activated (HVA) I_Ca_ in each cell was determined by stepping the membrane potential from a holding potential of -90 mV to between -60 and 30 mV, for 10 ms, in 10 mV increments. Following this procedure, the test current was evoked (-80 to 0 mV) every 30 s and monitored for current stability before drugs were applied. Cells were exposed to the conopeptides at increasing concentrations via a series of flow pipes positioned above the cells. All conopeptides were synthesized and analyzed as described previously [12]. The inhibition by conopeptides was quantified by measuring the current isochronically from the peak of the control current in the presence and absence of the drug.

### Induction of inflammatory pain

Mice were injected subcutaneously in the plantar surface of the left hindpaw with 50 μL undiluted Complete Freund’s Adjuvant (CFA) (Sigma-Aldrich, USA) under brief isoflurane anaesthesia (4% in air) using a 50 μL Hamilton syringe.

### Hindpaw weight bearing (incapacitance) and automated von Frey testing

Measurement of hindpaw weight bearing in mice provides a robust measure of severity of mechanical hyperalgesia following hindpaw inflammation and is sensitive to antinociceptive drugs [22]. Weight bearing was measured using a Linton incapacitance tester (Linton Instruments, UK). At each time point, five measurements were collected over 3–5 minutes, averaged and the ratio of load on left/right paw recorded as a single data point. Data points not involving clear contact of both paws with the test plates were not collected or analysed. Highly variable data were observed when incapacitance ratios when post-CFA ratios were much greater than 0.6. This suggested inadequate development of allodynia unless post-CFA ratios were less than 0.65. All animals exhibiting a post-CFA ratio of greater than 0.64 were therefore not tested further. In preliminary experiments, allodynia was also measured using an automated von Frey threshold tester (Dynamic Plantar Anesthesiometer, Ugo Basile). In this test mice were acclimatized to a raised, wire mesh floored chamber and the test was performed 5 times on each hindpaw over a period of approximately 5–8 minutes. The ratio of latency is calculated for each measurement and expressed as a ratio of left/right paw.

### Drug injection

Three days after induction of inflammation, each peptide injection was prepared by an independent researcher in sterile, pyrogen free isotonic saline (0.9%) vehicle and coded. A baseline measure of incapactiance was recorded immediately before drug injection. Immediately after baseline measurement, drugs or vehicle were injected by the observer who was blinded to the treatment subcutaneously near the scruff of the neck in a volume of 10 ml/kg. Each syringe used contained no more than 2 coded doses (sometimes 1) and most daily experiments included 8 animals. Incapacitance scores were then recorded for each animal at 1, 2, 4, and 6 hours after injection.

### Side effect scores

ω-Conotoxins produce characteristic side effect signs in rodents after intrathecal injection [5-7]. These include tail writhing, paw tremor and whole body shakes. All animals were observed individually for these signs and any other observable signs such as obvious sedation or hyperactivity, any form of shaking, display of discomfort including pilorection and escape behaviour. These were scored for each animal in an observation chamber for a period of 5 minutes at each time point.

### Motor side effects

Potential motor side effects were assessed in male C57Bl/6j mice (n = 30, 20–25 g). Animals were tested 2 hours after injection of either 2 mg/kg (0.2 mL, s.c.) of ω-conotoxin (CVID, CVIE, or CVIF) or saline. The accelerating rotarod (IITC Life Sciences, Series 8) rotational speed was increased from 5 to 45 rpm over 2 minutes. Time to fall (s) and peak rotational speed (rpm) were recorded, with at least 5 min inter-trial interval allowed. Mice were trained for 5 trials per day for the two days preceding testing. On testing day, 3 trials were recorded and averaged for each mouse Forepaw grip strength was determined using a grip strength meter (DFIS-2 series digital force gague, Columbus Instruments, Columbus, Ohio). The amount of force required to pull the mouse from a pull bar was recorded. Each mouse was tested 5 times, with 1 min inter-trial interval. The average peak tension (g), and tension normalised to body weight were compared.

### Data analysis

For electrophysiological analyses, all data are expressed as the mean ± SEM unless otherwise indicated. Concentration–response data were pooled for each group and fitted to a logistic equation with a minimum constrained to zero using the software package GraphPad Prism v. 5. Where noted, significant differences between means were tested using paired or unpaired two-tailed Student’s *t* tests, or when more than two groups were included, one-factor ANOVA, followed by Bonferroni's test. Differences between frequency data were tested using χ^2^ tests.

In behavioural experiments, after breaking the code for each drug treatment, data were collated and analysed using GraphPad Prism version 5. Raw incapacitance data for each peptide was analyzed by two-way ANOVA with repeated measures and, when significant, Bonferroni’s post-hoc tests for planned contrasts between vehicle and drug effects. To compare dose–response data and compare each group directly with vehicle controls, area under the time response curve was calculated for each animal at each dose of each peptide (and vehicle). One way ANOVA (ungrouped data) for each peptide was performed using the vehicle control group follow by Dunnett’s post-hoc test for differences from the vehicle group. The area under the curve (AUC) for dose–response data for all peptide were also compared by two factor ANOVA (ungrouped data) to test whether there were potency significant potency differences. Significant effects are shown throughout as * = P < 0.05, ** = P < 0.01, *** = P < 0.001.

### Drugs and chemicals

Bovine serum albumin (BSA), trypsin inhibitor (chicken egg white ovomucoid, Type II-O) and CdCl_2_ were from Sigma-Aldrich. Papain and collagenase were from Worthington Biochemical Corporation (Freehold, NJ, USA). ω-Conotoxins were provided by R.J. Lewis, Institute of Molecular Biology, University of Queensland. Nimodipine was fromTocris Cookson (Bristol, UK). ω-Agatoxin IVA and SNX-482 were from Peptide Institute, Inc. (Osaka, Japan).

## Abbreviations

CFA: Freund’s complete adjuvant; HBS: Hepes buffered saline; HVA: High voltage activated; VGCC: Voltage-gated calcium channel.

## Competing interests

RJL, PFA and MJC have filed the following provisional patent related to this manuscript: Novel ω-Conotoxins (CVIE). 2010 National Phase PCT/AU2010/001228 Lewis, Alewood, Drinkwater, Christie. The authors declare there are no other competing interests.

## Authors’ contributions

MS and SM carried out the mouse behavioural studies, performed statistical analysis and helped draft the manuscript. SSM carried out the electrophysiological recordings, performed statistical analysis and drafted the manuscript. RJL and PFA synthesized and provided the peptides, and participated in the design of the study. MJC designed and coordinated the study, and helped to draft the manuscript. All authors read and approved the final manuscript.
